# Determination of Atmospheric Pollen Grains by Volumetric Method in Sarıkamış District (Kars-Türkiye)

**DOI:** 10.3390/biology13070475

**Published:** 2024-06-26

**Authors:** Salih Akpınar, Mustafa Kemal Altunoğlu

**Affiliations:** Department of Biology, Faculty of Arts and Sciences, Kafkas University, 36100 Kars, Türkiye; mkaltun@gmail.com

**Keywords:** atmospheric pollen, pollen calendar, Sarıkamış

## Abstract

**Simple Summary:**

Among the aeroallergens present in the air that cause allergies, pollen holds a significant place. The majority of these pollen are dispersed by the wind. Numerous studies have been conducted in various regions of Türkiye and the world to determine the concentration of allergenic pollen in the atmosphere. The density of pollen in the atmosphere depends on meteorological factors, the flora of the region, altitude, and other similar factors. Therefore, the pollen concentration and the relationship of dominant pollen types with meteorological factors in the Sarıkamış region, one of the highest altitude areas in Türkiye and the world, have been studied. Based on the obtained data, a pollen calendar for the region has been prepared. Additionally, the main pollen seasons for pollen with high concentrations and the number of risky days for sensitive individuals have been identified.

**Abstract:**

Atmospheric pollen was investigated using the Lanzoni VPSS 2000 (Lanzoni, Bologna- Italy) device for 2 years between 2012 and 2013 in the Sarıkamış district of Kars province, one of the highest regions of Türkiye. A total of 37,909 pollen grains were collected: 15,298 pollen grains in 2012 and 22,611 pollen grains in 2013. Out of 43 identified taxa, 21 were arboreal, and 22 were non-arboreal. Pollen from arboreal plants accounted for 36.34% (13,778 pollen grains) of the total, while pollen from non-arboreal plants accounted for 63.56% (24,095 pollen grains). Additionally, 0.10% (36 pollen grains) belonged to unidentified pollen. The most frequent arboreal plant pollen in the Sarıkamış atmosphere were Pinaceae (29.79%), Cupressaceae/Taxaceae (2.54%), and *Morus* (1.30%). The main non-arboreal plant pollen in the atmosphere of Sarıkamış were Poaceae (44.60%), *Artemisia* (2.98%), Amaranthaceae (2.79%), *Rumex* (2.41%), Urticaceae (2.33%), *Plantago* (2.19%), and Boraginaceae (1.40%). The maximum pollen concentration was observed in June and was associated with the pollination of Pinaceae and Poaceae.

## 1. Introduction

Plants are pollinated in various ways, including hydrophily (water), anemophily (wind), ornithophily (birds), chiropterophily (bats), and entomophily (insects) [1]. While relatively few plants are wind-pollinated, pollen from these plants can cause diseases such as allergic rhinitis and allergic conjunctivitis in humans [2]. In recent years, pollen-related diseases have become a significant issue [3]. Aeropalynological studies aim to identify plants that cause allergies in humans.

Pollen concentration and diversity in a region are influenced by both the plant species within that region and those in surrounding areas. Pollen concentrations in the atmosphere fluctuate over time due to meteorological, ecological, and geographical factors [4,5,6,7,8]. Because pollen grains vary in size, they can induce various allergic reactions in humans. Typically, small-sized pollen grains trigger allergies by quickly entering the respiratory system and adhering to mucous membranes in the nasal cavity and trachea [9]. Allergic reactions are primarily triggered by proteins present in the pollen grains. Common allergic reactions caused by pollen include hay fever, asthma, rhinitis, and conjunctivitis [10]. Additionally, certain pollen proteins can cross-react with similar proteins in various foods, leading to oral allergy syndrome in some individuals [11].

Determining the types and concentrations of allergenic pollen in the atmosphere is crucial for understanding the health risks posed to individuals in specific regions. Knowing the pollen seasons and peak times of pollen levels helps sensitive individuals with pollen allergies take precautions against the pollen to which they are allergic. This study aims to identify allergenic pollen types and their concentrations in the atmosphere of Sarıkamış district in the Kars province, situated at an elevation of 2225 m, one of the highest places in Türkiye. Located within the Iran-Turan phytogeographic region, this area has not been previously studied aeropalynologically. Our objectives include determining the start and end dates of pollen seasons for dominant pollen, assessing the duration of pollen seasons, and identifying the number of days with heightened risk for susceptible individuals using volumetric methods. Additionally, we seek to examine the relationship between these findings and various meteorological factors such as daily temperature, wind speed, humidity, and total precipitation.

## 2. Materials and Methods

**Figure 1:**
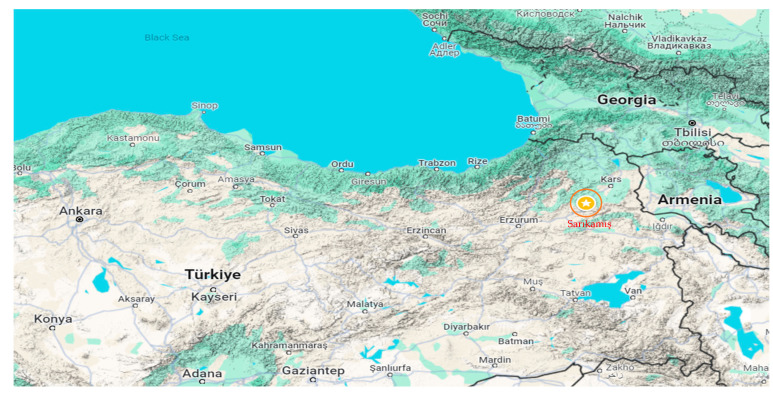
Location of the pollen monitoring site in Sarıkamış, Türkiye [15].

Sarıkamış (40°20′17″ N–42°34′23″ E) is located in the Erzurum-Kars section of the Eastern Anatolia Region and is surrounded by Kağızman (Kars) to the east, Eleşkirt (Ağrı) to the south, Horasan (Erzurum) to the southwest, Narman (Erzurum) to the west, Şenkaya (Erzurum) to the northwest, and Selim (Kars) to the north (Figure 1). It covers an area of 1751 km^2^, with 37,000 hectares covered by *Pinus slyvestris* forests. Its altitude above sea level is 2225 m. The main plant species include Pinaceae, *Quercus*, *Artemisia*, and Cupressaceae/Taxaceae [12]. Due to climatic conditions, agricultural cultivation is not practiced in the region, and naturally occurring plants are widespread.

In the region, genera and species belonging to the Pinaceae (*Pinus slyvestris* L.), Cupressaceae (*Juniperus oxycedrus* L., *Juniperus foetidissima* Willd., *Juniperus nana* Willd.), Caprifoliaceae (*Viburnum oriantale* Pall.), Betulaceae (*Betula nana* L.), Ericaceae (*Vaccinium myrtillus* L.), Saliceae (*Populus tremula* L., *Salix caprea* L.), Rosaceae (*Alchemilla sintenisii* Rothm., *Sorbus aucuparia* L., *Cerasus avium* (L) Moench, *Pyrus elaeagnifolia* Pall., *Rosa spinosissima* L., *Rosa dumalis* subsp. boisseri (Crep.) Ö. Nilsson, *Rubus caesius* L., Grossulariaceae (*Rihes uva-crispa* L.), Celastraceae (*Euonymus latifolius Mill.*), Asteraceae (*Artemisia absinthium* L., *Cichorium intybus* L., *Cirsium* sp., *Onopordum* sp., *Aster amellus* L., *Helichrysum plicatum* DC., *Artemisia austriaca* Jacq., *Tanacetum balsamita* L., *Achillea millefolium* L., *Cota tinctoria* (L.) J. Gay, *Centaurea glastifolia* L., *Lactuca serriola* L., Cyanus depressus (M. Bieb.) Soják, Fabaceae (Trifolium *trichopterum* Pančić, *Trifolium pratense* L., *Trifolium aereum* Pollich, *Trifolium repens* L, *Lotus corniculatus* L., *Vicia hirsuta* (L.) Gray, *Astragalus* sp., *Securigera orientalis* subsp. *orientalis*, *Securigera varia* (L.) Lassen, *Melilotus officinalis* (L.) Desr., *Lathyrus roseus* Steven, *Spartium junceum* L., *Poaceae* (*Stipa pontica* P.A. Smirn., *Koeleria pyramidata* (Lam.) P. Beauv., *Dactylis glomerata* L., *Festuca chalcophaea* V. Krecz. & Bobrov, *Melica persica* Kunth, *Melica* sp. *Elymus hispidus* subsp. *hispidus*, *Agrostis stolonifera* L., *Arrhenatherum elatius* subsp. *elatius*, *Bromus inermis* Leyss., *Bromus* sp., *Bromus tectorum* L., *Dactylis glomerata* L., *Festuca airoides* Lam., *Koeleria* sp., *Phleum montanum* K. Koch, *Poa bulbosa* L. *Poa pratensis* L. *Poa* sp., Brassicaceae: *Lepidium campestre* (L.) Aiton, *Alyssum murale* Waldst. & Kit., Boraginaceae *Onosma* sp., *Echium vulgare* L., Campanulaceae *CampanuIa rapunculoides* L., *Campanula glomerata* L., Plantaginaceae: *Plantago lanceolata* L., *Plantago major* L., Apiaceae: *Astrodaucus orientalis* (L.) Drude, *Ferula orientalis* L., Lamiaceae: *Ajuga chamaepitys* (L.) Schreb, *Sideritis montana* L., Papaveraceae: *Papaver fugax* Poir., Caryophyllaceae: *Silene compacta* Fisch. Ex. Hornem., Polygonaceae: *Rumex acetosella* L., Cistaceae: *Helianthemum Oelandicum* (L.) DC., Hypericaceae: *Hypericum perforatum* L., Onagraceae: *Epilobium angustifolium* L., Dipsacaceae: *Cephalaria sparsipilosa* V.A. Matthews, Scrophulariaceae: *Verbascum oreophilum* K. Koch, Ranunculaceae: *Ranunculus grandiflorus* L. Among these, the species *Cephalaria sparsipilosa* V.A. Matthews, *Verbascum oreophilum* K. Koch, *Alchemilla sintenisii* Rothm., and *Papaver fugax* Poir. are endemic and categorized as of least concern, while *Trifolium trichopterum* Pančić is identified as a rare species but not endemic. [13]. *Elymus hispidus* subsp. *hispidus*, *Agrostis stolonifera* L., *Arrhenatherum elatius* subsp. *elatius*, *Bromus inermis* Leyss., *Bromus* sp., *Bromus tectorum* L., *Dactylis glomerata* L., *Festuca airoides* Lam., *Koeleria* sp., *Phleum montanum* K. Koch, *Poa bulbosa* L. *Poa pratensis* L., and *Poa* sp. Taxa constitute more than half of the pasture areas [14].

**Figure 2:**
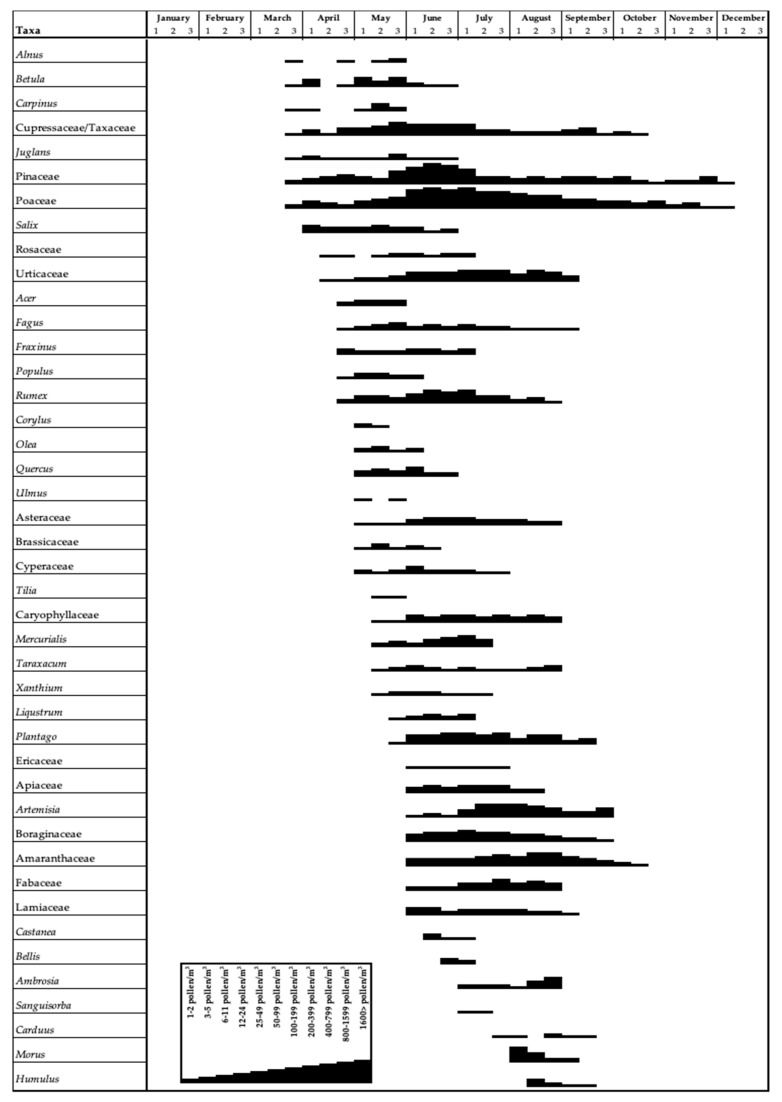
Pollen calendar of Sarıkamış (years 2012–2013).

In the study conducted using the volumetric method, a Hirst-type volumetric pollen trap (Lanzoni VPPS 2010) was employed. The pollen trap was positioned on the roof of a building approximately 15 m high in the center of Sarıkamış, and samples were collected between 1 January 2012, and 31 December 2013. Weekly samples were brought to the laboratory and converted into daily preparations by dividing them into 7 equal parts of 48 mm The daily and hourly average concentration of the number of pollen grains was defined by hourly counting at 400× magnification under a Leica light microscope at intervals of 2 mm (representing 1 h), and their abundance in 1 m^3^ of air was measured following the methodology recommended by the REA (Spanish Aerobiological Network) [16]. Daily variations and the main pollen seasons were determined using Andersen’s method [17]. The start of the Main Pollen Season (MPS) was defined as the day when the daily pollen count reached 2.5% of the annual pollen index (API), and it ended when 97.5% of the annual pollen count was reached. Threshold values for daily pollen concentrations in the air affecting sensitive individuals were calculated according to the REA standards [16]. A pollen calendar was constructed using the Spieksma model [18]; daily pollen counts from 10-day periods over the study years were summed and averaged to prepare the pollen calendar for Sarıkamış (Figure 2). Average pollen counts were correlated with parameters such as temperature, wind speed, relative humidity, and total precipitation obtained from the Turkish Meteorological Directorate (40°19′58″ N–42°35′53″ E). The relationship between meteorological factors and pollen counts was evaluated using Spearman’s correlation test in SPSS 20.

## 3. Results

A total of 43 taxa were identified in the atmosphere of Sarıkamış throughout the two-year study. There were 21 arboreal plants and 22 non-arboreal plants out of 43 taxa. A total of 37,909 pollen grains were counted: 15,298 pollen grains in 2012 and 22,611 pollen grains in 2013. 33.83% (13,778 pollen) of the total pollen amounts belonged to arboreal plants, 63.56% (24,095 pollen) to non-arboreal plants, and 0.09% (36 pollen) to unidentified plants. Tables show the monthly pollen amounts of arboreal and non-arboreal plants throughout the two-year study (Table 1, Figure 2). It was determined that pollen was observed in the atmosphere from March to December. 92.51% of the total pollen was found in June, July, and August. The highest taxon diversity was observed in June, with 32 taxa, and 53.83% of the total pollen was recorded in June. In both years, arboreal plants were heavily found in the atmosphere in May and June, and non-arboreal plants in June and July. The counts of pollen species in the atmosphere were recorded in the form of a pollen calendar (Figure 2). The most common pollen species of arboreal plants found in the atmosphere of Sarıkamış were Pinaceae (29.79%; 11,292 pollen), Cupressaceae/Taxaceae (2.54%; 961 pollen), and *Morus* (1.30%; 492 pollen). These accounted for 33.62% of the total pollen. Non-arboreal plants that abound in Sarıkamış atmosphere were Poaceae (44.60%, 16,908 pollen), *Artemisia* (2.98%, 1130 pollen), Amaranthaceae (2.79%, 1056 pollen), *Rumex* (2.41%, 912 pollen), Urticaceae (2.33%, 884 pollen), *Plantago* (2.19%, 832 pollen) and Boraginaceae (1.40%, 531 pollen), which accounted for 58.70% of the total pollen. Table 1 presents the densities of taxa in the atmosphere during 2012–2013.

The majority of the pollen detected in the atmosphere was from the Poaceae family of plants, also known as grasses, constituting 44.60% of the total pollen concentration (Table 1). The maximum concentrations of Poaceae pollen were observed in June (20.65%) and July (19.88%) (Figure 2). The main pollen season lasted for 91 days from 4 June to 31 August in 2012 and for 73 days from 4 June to 16 August in 2013. It was determined that there were 26 risky days for sensitive individuals in 2012 and 36 risky days in 2013 (Table 2).

The arboreal taxon with the highest concentration was the Pinaceae family, comprising 29.79% of the total pollen count and reaching a maximum concentration in June (26.66%) (Table 1, Figure 2). The main pollen season spanned 43 days from 30 May to 12 July in 2012 and 45 days from 1 June to 16 July in 2013. It was found that the number of high-risk days for sensitive individuals was 16 and 27 in 2012 and 2013, respectively (Table 2).

The third highest taxon, *Artemisia*, contributed 2.98% of the total pollen and displayed its peak concentration during July (1.50%) and August (1.22%) (Table 1, Figure 2). The main pollen season spanned 79 days from 11 July to 28 August in 2012 and 75 days from 10 July to 23 August in 2013. The duration of risk exposure for individuals with pollen allergies was identified as 3 days in 2012 and 4 days in 2013 (Table 2).

Amaranthaceae accounted for 2.79% of the total pollen, with the maximum density occurring in August (1.55%) (Table 1, Figure 2). The main pollen season was found to last for 99 days between 14 June and 21 September in 2012, and for 94 days between 9 June and 11 September in 2013. When we look at the number of risky days for sensitive individuals, there were 3 days in 2012 and 1 day in 2013 (Table 2).

Cupressaceae/Taxaceae constituted 2.54% of the total pollen and showed peak concentrations in May (0.86%) and June (0.94%) (Table 1, Figure 2). The primary pollen season for this taxon extended for 120 days, from 3 April to 1 August in 2012, and for 140 days, from 24 April to 11 September in 2013. No risk period was observed in 2012, but one day with a risk of exposure to allergenic pollen was identified in 2013 (Table 2).

*Rumex* represented 2.41% of the total pollen, with the highest concentration occurring in June (1.31%) (Table 1, Figure 2). The main pollen season was determined to last 91 days between 2 May and 1 August in 2012 and 73 days between 30 May and 25 July in 2013. The number of days considered risky for susceptible individuals was 1 in 2012 and 2 in 2013 (Table 2).

Urticaceae constituted 2.33% of the total pollen, with a peak concentration in July (1.06%) (Table 1, Figure 2). The main pollen season was identified to span over 96 days from 23 May to 27 August in 2012, and 90 days from 29 May to 27 August in 2013. The number of days posing a risk to susceptible individuals was eight in 2012 and one in 2013 (Table 2).

*Plantago* accounted for 2.19% of the total pollen and exhibited the highest concentrations in June (0.78%) and July (0.92%) (Table 1, Figure 2). The main pollen season persisted for 96 days between 8 June and 12 September 2012, and for 87 days between 3 June and 29 August 2013. No days were detected as being risky for sensitive individuals in 2012, whereas one day was detected in 2013 (Table 2).

Boraginaceae constituted 1.40% of the total pollen, with the highest concentration observed in July (0.65%) (Table 1, Figure 2). The main pollen season lasted for 82 days between 10 June and 31 August in 2012 and for 75 days between 9 June and 23 August in 2013. The number of days with a risk of allergic symptoms for sensitive individuals was found to be 1 in 2012 and 9 in 2013 (Table 2).

*Morus* was responsible for 1.30% of the overall pollen. Its peak concentration occurred in August (1.28%) (Table 1, Figure 2). The main pollen season spanned 13 days between 9 August and 22 August in 2012 and 21 days between 7 August and 28 August in 2013. The number of days posing a risk to individuals with hypersensitivity was established as three in 2012 and four in 2013 (Table 2).

Meteorological data, including mean temperature, mean relative humidity, mean wind speed, and total precipitation for 2012 and 2013, are presented in Table 3. No significant differences were observed in the meteorological factors between the two years. During the two-year study, the monthly mean temperature ranged between −9.9 °C and 17.4 °C. Pollen was not detected in the atmosphere during most of January, February, and March, when the temperature dropped below 0 °C. The mean relative humidity fluctuated between 51.9% and 81.2%, with an annual average of 66.1%. Total precipitation ranged from 0 mm to 74.6 mm. Mean wind speed varied between 1 m/s and 3.7 m/s, with a significant increase observed only in July 2013 (Table 3).

In this study, the relationship between the daily total pollen amounts of the taxa Amaranthaceae, *Artemisia*, Boraginaceae, Cupressaceae/Taxaceae, *Morus*, *Plantago*, Pinaceae, Poaceae, *Rumex*, and Urticaceae, which were among the most abundant taxa in the atmosphere of Sarıkamış, Kars province in 2012 and 2013, and meteorological factors including daily mean temperature, daily mean relative humidity, daily mean wind velocity, and daily mean precipitation was investigated using Spearman’s correlation analysis (Table 4). According to the analysis of the average data obtained during 2012 and 2013, a statistically significant positive correlation (*p* < 0.01) was found between the daily total pollen count, temperature, and wind speed. A statistically significant negative correlation (*p* < 0.01) was detected between the daily total pollen count and relative humidity. No significant correlation was found between the daily pollen count and precipitation. A positive correlation (*p* < 0.01) was determined between Poaceae, Pinaceae, Urticaceae, Amaranthaceae, *Artemisia*, and *Rumex* pollen counts and temperature. A statistically significant positive correlation (*p* < 0.05) was found between the *Plantago* pollen counts and temperature. No significant correlation was found between Cupressaceae/Taxaceae, *Morus*, and Boraginaceae pollen counts and temperature. A positive correlation (*p* < 0.01) was found between the *Plantago* and Boraginaceae pollen counts and relative humidity, and a negative correlation (*p* < 0.01) was found between the daily pollen count and Cupressaceae/Taxaceae pollen counts and relative humidity. A statistically significant positive correlation (*p* < 0.01) was detected between wind speed and Poaceae, Urticaceae, and *Artemisia* pollen counts. A negative correlation (*p* < 0.05) was found between *Artemisia* pollen counts and precipitation (Table 4).

## 4. Discussion

This study aimed to characterize the pollen types present in the atmosphere of Sarıkamış, Türkiye’s highest-altitude region, utilizing Lanzoni VPSS during the years 2012–2013. A comprehensive analysis identified a total of 43 taxa. Unlike many other aeropalynological studies conducted both in Türkiye and globally, this study observed a predominance of pollen grains from non-arboreal plants, constituting 59.71% of the total pollen density. This dominance can be attributed to the specific vegetation type, climatic conditions, and geographical structure of the Sarıkamış area. Comparative studies conducted elsewhere have also highlighted similar trends favoring non-arboreal plant pollen dominance. For instance, densities of 59.28% in Bitlis [19], and 63.57% in Kars, Türkiye [20], 83% in Jaipur, India [21], 60.3% in Leiden, Netherlands [22], 55% in Trentino, Italy [23], and 52% in Porto, Portugal [24] have been reported. These findings underscore the variability in pollen distribution, which is influenced by local environmental factors across different regions worldwide.

The pollen concentrations, pollen calendar, average concentrations of dominant pollen over two years, the main pollen seasons of dominant pollen, and the number of risky days for allergic patients are detailed in tables for both arboreal and non-arboreal plants. Due to temperatures dropping below 0 °C, no pollen was detected in the atmosphere during January and February. However, pollen began to appear at the end of March as the temperature rose. The dominant arboreal plant pollen types were Pinaceae (29.79%), Cupressaceae/Taxaceae (2.54%), and *Morus* (1.30%). The concentration of arboreal plant pollen began increasing in May and peaked in June in regions such as Mardin-Kızıltepe [25] and Kayseri [26], primarily due to the high density of Pinaceae and Cupressaceae/Taxaceae taxa. Arboreal plant pollen was detected in the atmosphere, albeit at lower levels, until December. Similarly, pollen from non-arboreal plants increased in May, peaked in June, and remained present in the atmosphere until the end of the year. The dominant non-arboreal taxa included Poaceae (44.60%), *Artemisia* (2.98%), Amaranthaceae (2.79%), *Rumex* (2.41%), Urticaceae (2.33%), *Plantago* (2.19%), and Boraginaceae (1.40%).

Poaceae emerges as the predominant taxon in our study area, constituting a significant trigger for allergic reactions globally. With its vast distribution and wind-pollination mechanism spanning approximately 11,500 species, Poaceae presents a formidable challenge for allergy sufferers due to the difficulty in distinguishing its pollen variants [27]. Our findings align with previous studies, showcasing Poaceae’s dominance in regions such as Bitlis (25.19%) [19], Mardin (21.21%) [28], and Van (20.94%) [4], underscoring its pervasive presence across diverse geographical landscapes. Moreover, comparably high densities of Poaceae pollen have been documented worldwide, including notable figures such as 31.3% in Buenos Aires, Argentina [29], 32.85% in Lagos, Nigeria [30], 39.5% in Lugo, Spain [31], and 42% in Jaipur, India. [21]. This underscores the global impact of Poaceae as a major contributor to allergic diseases associated with pollen allergies. The extended flowering and pollination period of Poaceae, lasting an average of 70 days, poses a prolonged risk to sensitive individuals. Over the course of our two-year study, we identified 62 risky days, further emphasizing the challenges faced by those susceptible to Poaceae-induced allergic reactions.

*Artemisia*, with a concentration of 2.98% identified in the present study, demonstrates a consistent presence similar to data from studies in Ardahan [32], Isparta [33], and Trabzon [34] within Türkiye, as well as globally in places such as Leiden, Holland [22], Hungary [35], and Delhi, India [36]. The prevalence of *Artemisia* pollen during the summer months is a common trend observed in both European and Turkish studies, indicating a seasonal pattern that aligns with our findings. *Artemisia* is known for its allergenic properties, often contributing to late summer and autumn hay fever. The high concentrations observed during these periods can exacerbate the symptoms in individuals with pollen allergies. This correlation between seasonal spikes in *Artemisia* pollen and increased allergic reactions underscores the importance of monitoring and managing this pollen type, particularly in regions where it is prevalent. Additionally, the consistency of *Artemisia* pollen concentrations across diverse geographical locations highlights its widespread impact on public health, necessitating further research and preventive measures to mitigate its effects on allergy sufferers.

During summer and autumn, Amaranthaceae pollen, recognized as one of the most significant allergens, was reported as the dominant pollen type in the regions of Aksaray [37], Afyon [38], Tekirdağ [39], Konya [40], and Van [4] in Türkiye. In Sarıkamış, *Rumex* and Urticaceae were identified, with densities exceeding 2%. Similar densities for these taxa have been reported in Madrid [41], Vigo [42], and Lugo [31] in Spain, as well as in Bitlis [19], Van [4], Trabzon [34], and Tekirdağ [39] in Türkiye. The highest concentration of *Rumex* was observed in June, while Urticaceae reached its peak density in July. The high levels of Amaranthaceae pollen, particularly in summer and autumn, indicate its significant role in allergic reactions during these seasons. Its widespread presence in various regions underscores its importance as an allergen and highlights the need for effective monitoring and public health strategies to mitigate its impact. Similarly, the notable presence of *Rumex* and Urticaceae pollen suggests that these taxa are also important contributors to airborne allergens in the region.

Although *Plantago* is considered an important allergen in many countries, its allergic effect is not fully understood because its flowering period overlaps with that of the Poaceae family, leading to an increase in symptoms during the same period. In the study area, *Plantago* pollen was recorded intensively in June and July, mirroring the pattern observed for Poaceae. High densities of *Plantago* pollen were found in studies conducted in the Iran-Turan Region, including Bitlis [19], Van [4], Aksaray [37], Konya [39], Mardin [28], and Elazığ [43]. Similar findings were reported in other parts of the world, such as El Hadjar, Algeria [44]; Bratislava, Slovakia [45]; and Cordoba, Spain [46]. The concurrent flowering periods of *Plantago* and Poaceae complicate the precise attribution of allergic reactions to each taxon, underscoring the need for detailed phenological studies to determine their individual contributions to allergic responses.

The seasonal peaks of this pollen—*Rumex* in June and Urticaceae in July—point to critical periods when individuals with sensitivities may experience heightened symptoms. This temporal pattern suggests that allergy management and mitigation efforts should particularly focus on these months to help reduce the burden on affected individuals. Consistent findings across multiple regions reinforce the need for regional pollen monitoring and public health initiatives to address the impact of these allergens on respiratory health.

Boraginaceae pollen, for which little information is available regarding its allergic effects, was not found to be dominant in any study conducted in Türkiye. However, it was recorded as the dominant pollen in Funchal, Portugal [47] at 3.80% and in Calcutta, India [48] at 3.20%, similar to the findings of the present study. The limited information on Boraginaceae pollen’s allergic effects suggests an area for future research, especially given its recorded dominance in regions outside Türkiye. Understanding its potential impact on allergies could provide new insights into regional differences in pollen allergenicity and contribute to more comprehensive approaches for allergy prevention and management.

Pinaceae pollen, which exhibits the highest concentration among arboreal plant pollen, has been reported as the main pollen type in several regions, including Eskişehir-Sivrihisar (69.31%) [49], Kütahya (51.56%) [50], Eskişehir (48.13%) [51], Kastamonu (42.9%) [52], Ankara (32.43%) [53], Yunnan, China (38.7%) [54], Nerja (25.04%) [55], and Vigo (25.1%) [42] in Spain. In our study, the dominance of Pinaceae pollen aligns with these findings, further emphasizing its prevalence in diverse geographical areas. Although the main pollen season for Pinaceae lasts approximately five months each year, the total number of high-risk days identified was 43. This was primarily due to the peak concentration of Pinaceae pollen occurring in June. The extended flowering period of Pinaceae results in prolonged exposure of sensitive individuals, increasing the likelihood of allergic reactions. The widespread distribution and prolonged pollen season of Pinaceae highlight the importance of monitoring this pollen type, particularly for allergy management and mitigation strategies. This consistent pattern across various regions suggests that Pinaceae pollen is a significant contributor to seasonal allergies and necessitates focused attention in public health planning and allergy forecasting.

Cupressaceae/Taxaceae, recognized as a significant allergen, was identified as the second most dominant arboreal plant pollen type in Türkiye. This pollen type has primarily been recorded as the leading pollen type in various regions, including Adana [56], Hatay [57], Antalya [58], Istanbul [59], and Aydın-Kuşadası [60]. The widespread presence of Cupressaceae/Taxaceae pollen underscores its importance in contributing to seasonal allergic reactions.

*Morus* pollen, known to cause moderate allergic reactions, was detected at a considerable density in Sarıkamış. The prevalence of *Morus* pollen in Sarıkamış aligns with similar findings from other regions such as Van [4], Bitlis [19], Afyon [38], and Kütahya [50] in Türkiye, as well as internationally in locations like Kazakhstan, Bishkek [61], Canada, Toronto [62], and Taiwan, Taichung [63]. The consistent detection of *Morus* pollen in these areas suggests its widespread distribution and potential impact on individuals with allergies.

The identification of Cupressaceae/Taxaceae and *Morus* as prominent pollen types highlights the need for continuous monitoring and effective allergy management strategies. Understanding the distribution and concentration of these pollen types can aid in developing targeted approaches to mitigate allergic reactions, particularly during the peak pollen seasons.

Previous studies (Van, Türkiye [4], Bitlis, Türkiye [19], Kars, Türkiye [20], Jaipur, India [21], Leiden, Holland [22], Trentino, Italy [23], Mardin, Türkiye [28], Konya, Türkiye [40], Konya, Türkiye [40], Madrid, Spain [41], and Fucnhal City, Portugal [47]) conducted in various regions have consistently shown that Poaceae, Pinaceae, *Artemisia*, Amaranthaceae, Cupressaceae/Taxaceae, *Rumex*, Urticaceae, *Plantago*, Boraginaceae, and *Morus* are dominant pollen types (Table 5).

In the two-year study, the duration of pollen seasons for allergenic pollen ranged from 13 to 140 days. The total number of risky days for dominant pollen was found to be 147. Notably, 92.51% of the total pollen count was observed during the summer months. This high concentration during summer can be attributed to the local climate conditions; specifically, temperatures are below 0 °C during the winter months, preventing pollen release, and then rise sharply as summer approaches, creating optimal conditions for pollen production and dispersal.

Based on statistical analyses, significant correlations were identified between meteorological parameters and pollen concentrations. Generally, it is stated that temperature and wind increase pollen concentrations, while humidity and rainfall decrease them [31,45,64,65,66]. However, in this study, both consistent and contradictory results were observed. Temperature exhibited a positive correlation with pollen concentrations from taxa other than the dominant taxa, such as Cupressaceae/Taxaceae, Boraginaceae, and *Morus*. Various studies have identified positive correlations between daily temperature and pollen concentrations of Poaceae, Pinaceae, Urticaceae, Amaranthaceae, *Artemisia*, *Rumex*, and *Plantago*, including those conducted in Gümüşhane (Pinaceae, Poaceae) [67], Hatay (Pinaceae, Poaceae, Urticaceae) [57], Sinop (Amaranthaceae, Pinaceae, *Plantago*, Poaceae) [68], Van (Poaceae, *Plantago*) [4], Mexico City (Poaceae, Urticaceae) [69], Alexandroupolis (Poaceae, Amaranthaceae) [70], Toledo (Amaranthaceae, Pinaceae, Poaceae, Urticaceae) [71], and Porto (Urticaceae, Poaceae, *Plantago*, *Rumex*, Amaranthaceae) [24]. This correlation suggests that significant temperature increases influence the release and spread of these pollen types, leading to higher concentrations in the atmosphere during warmer periods. This finding aligns with the hypothesis that climate and temperature play crucial roles in pollen season dynamics and the resultant impact on allergy sufferers.

These results emphasize the importance of monitoring pollen levels and temperature fluctuations to predict allergy seasons and prepare effective health advisories. Future research should further investigate the specific mechanisms by which temperature and other climatic factors influence pollen production and distribution, potentially leading to more precise forecasting and better management strategies for pollen-related allergies. Similar to findings in many studies, our research also revealed a positive correlation between pollen concentrations of Poaceae, Pinaceae, Urticaceae, Amaranthaceae, *Artemisia*, *Rumex*, *Plantago*, and daily temperature. Furthermore, a negative correlation was recorded only between relative humidity and Cupressaceae/Taxaceae. Similar results have been observed in studies conducted in Van [4], Konya [40], and Mexico City [69]. Contrary to many previous studies, the pollen concentrations of *Plantago* and Boraginaceae increased with increasing relative humidity. This is thought to be due to the pollen seasons coinciding with periods of increased relative humidity. Wind speed showed a positive correlation with Poaceae, Urticaceae, and *Artemisia*. Similar results have been seen in studies conducted in Hatay [57], Sinop [68], Konya [40], Mexico City [69], and Porto [24]. Several studies have demonstrated a negative relationship between rainfall and pollen concentration. In our study, a negative relationship was observed only between *Artemisia* pollen and rainfall.

## 5. Conclusions

This two-year study has identified a total of 37,909 pollen grains per cubic meter belonging to 21 arboreal and 22 non-arboreal plant taxa in Sarıkamış, Türkiye. In contrast to many similar studies conducted in Türkiye, non-arboreal plant pollen was found to be abundant, while arboreal plant pollen was scarce. This disparity can be attributed to several factors specific to the study area: the prevalence of low temperatures during winter and late autumn, the high altitude of the region (above 2000 m), and the minimal temperature variations between day and night throughout the year.

As a result of this study, detailed information on the pollination periods and daily, monthly, and yearly pollen counts of significant allergens in Sarıkamış has been documented. A pollen calendar specific to Sarıkamış has been compiled, which is expected to be valuable for individuals sensitive to pollen allergies, as well as for visitors and allergy specialists in the region. The data obtained from this research provide insights into pollen dynamics in a high-altitude environment with specific climatic conditions, contributing to a better understanding and management of pollen-related allergies in Sarıkamış and similar regions.

## Figures and Tables

**Table 1:** Annual pollen counts and percentage of pollen taxa recorded in the Sarıkamış atmosphere (2012–2013).

		2012		2013		Mean	
	Taxa	Total	%	Total	%	Total	%
Arboreal plants	Pinaceae	4973	32.51	6319	27.95	5646	29.79
	Cupressaceae/Taxaceae	472	3.09	489	2.16	481	2.54
	*Morus*	197	1.29	295	1.30	246	1.30
	*Betula*	41	0.27	167	0.74	104	0.55
	*Quercus*	71	0.46	72	0.32	72	0.38
	*Salix*	80	0.52	54	0.24	67	0.35
	*Fagus*	47	0.31	71	0.31	59	0.31
	*Fraxinus*	42	0.27	39	0.17	41	0.21
	*Carpinus*	2	0.01	63	0.28	33	0.17
	*Acer*	49	0.32	0	0.00	25	0.13
	*Populus*	42	0.27	4	0.02	23	0.12
	Rosaceae	20	0.13	14	0.06	17	0.09
	*Liqustrum*	17	0.11	23	0.10	20	0.11
	*Juglans*	17	0.11	20	0.09	19	0.10
	*Olea*	23	0.15	4	0.02	14	0.07
	Castanea	16	0.10	1	0.00	9	0.04
	*Alnus*	1	0.01	10	0.04	6	0.03
	Ericaceae	5	0.03	5	0.02	5	0.03
	*Corylus*	9	0.06	0	0.00	5	0.02
	*Tilia*	2	0.01	0	0.00	1	0.01
	*Ulmus*	2	0.01	0	0.00	1	0.01
	Arboreal plants	6128	40.06	7650	33.83	6889	36.34
Non-arboreal plants	Poaceae	5482	35.83	11,426	50.53	8454	44.60
	*Artemisia*	492	3.22	638	2.82	565	2.98
	Amaranthaceae	568	3.71	488	2.16	528	2.79
	*Rumex*	452	2.95	460	2.03	456	2.41
	Urticaceae	579	3.78	305	1.35	442	2.33
	*Plantago*	284	1.86	548	2.42	416	2.19
	Boraginaceae	216	1.41	315	1.39	266	1.40
	Fabaceae	256	1.67	119	0.53	188	0.99
	*Mercurialis*	217	1.42	129	0.57	173	0.91
	Caryophyllaceae	117	0.76	110	0.49	114	0.60
	Apiaceae	75	0.49	123	0.54	99	0.52
	Asteraceae	97	0.63	80	0.35	89	0.47
	Lamiaceae	51	0.33	98	0.43	75	0.39
	*Ambrosia*	46	0.30	38	0.17	42	0.22
	Cyperaceae	47	0.31	32	0.14	40	0.21
	*Taraxacum*	34	0.22	34	0.15	34	0.18
	*Humulus*	35	0.23	4	0.02	20	0.10
	*Bellis*	32	0.21	0	0.00	16	0.08
	Brassicaceae	27	0.18	0	0.00	14	0.07
	*Xanthium*	18	0.12	6	0.03	12	0.06
	*Carduus*	8	0.05	5	0.02	7	0.03
	*Sanguisorba*	2	0.01	2	0.01	2	0.01
	Non-arboreal plants	9135	59.71	14,960	66.16	12,048	63.56
	Unidentified	35	0.23	1	0.00	18	0.09
	Total	15,298	100.00	22,611	100.00	18,955	100.00

**Table 2:** Characteristics of the main pollen season (MPS) for the most essential taxa (start and end dates), season length (days), and maximum values of monthly pollen concentration (pollen/m^3^).

		2012	2013
Poaceae	Main pollen season	1 June—31 August	4 June—16 August
	Main pollen season length (days)	91	73
	Maximum daily pollen/m^3^	12 July—358	1 July—719
	Number of days with risk	26	36
Pinaceae	Main pollen season	30 May—12 July	1 June—16 July
	Main pollen season length (days)	43	45
	Maximum daily pollen/m^3^	17 June—812	27 June—687
	Number of days with risk	16	27
*Artemisia*	Main pollen season	11 July—28 September	10 July—23 September
	Main pollen season length (days)	79	75
	Maximum daily pollen/m^3^	27 July—35	16 July—38
	Number of days with risk	3	4
Amaranthaceae	Main pollen season	14 June—21 September	9 June—11 September
	Main pollen season length (days)	99	94
	Maximum daily pollen/m^3^	12 August—34	28 August—26
	Number of days with risk	3	1
Cupressaceae/Taxaceae	Main pollen season	3 April—1 August	24 April—11 September
	Main pollen season length (days)	101	140
	Maximum daily pollen/m^3^	18 May—31	27 May—102
	Number of days with risk	-	1
*Rumex*	Main pollen season	2 May—1 August	30 May—25.07
	Main pollen season length (days)	91	56
	Maximum daily pollen/m^3^	17 June—33	1 July—87
	Number of days with risk	1	2
Urticaceae	Main pollen season	23 May—27 August	29 May—27 August
	Main pollen season length (days)	96	90
	Maximum daily pollen/m^3^	22 July—26	18 July—18
	Number of days with risk	8	1
*Plantago*	Main pollen season	8 June—12 September	3 June—29 August
	Main pollen season length (days)	96	87
	Maximum daily pollen/m^3^	11 July—10	1 July—34
	Number of days with risk	-	1
Boraginaceae	Main pollen season	10 June—31 August	9 June—23 August
	Main pollen season length (days)	82	75
	Maximum daily pollen/m^3^	26 July—12	18 June—22
	Number of days with risk	1	9
*Morus*	Main pollen season	9 August—22 August	7 August—28 August
	Main pollen season length (days)	13	21
	Maximum daily pollen/m^3^	9 August—39	11 August—42
	Number of days with risk	3	4

**Table 3:** Mean monthly meteorological data recorded in Sarıkamış, obtained from the State Meteorological Service of Türkiye.

Meteotological Data	Year	Jan.	Feb.	Mar.	Apr.	May	Jun.	Jul.	Aug.	Sep.	Oct.	Nov.	Dec.	Annual Average
Monthly Average Relative Humidity (%)	2012	78.9	73.3	68.9	68.3	71.1	57.4	65.6	51.9	56.9	70.9	76.5	81.2	64.5
	2013	75	79.2	70.1	65.7	65.8	64.2	65.6	61.8	55	60.6	77	71.1	67.6
Monthly Average Wind Speed (m/sn)	2012	1.4	1.3	1.9	1.6	1.2	2.5	3.7	1.6	1	0.9	1.1	1.2	1.6
	2013	1.5	1.3	1.9	1.5	1.5	1.2	1.3	1.1	1.1	1.2	1.1	1.2	1.3
Monthly Average Temperature (°C)	2012	−7.3	−9.9	−6.1	5.5	9.3	14.1	11.8	17.4	12.7	7.5	2.7	−5.6	4.3
	2013	−7.3	−5	−1.2	5.1	9.3	12.6	15.7	15.3	11.8	4.4	1.6	−9.7	4.4
Monthly Total Precipitation (mm)	2012	20.8	36.8	14	26.6	40	19	0.2	1.4	16.6	36.2	3.6	0	17.9
	2013	0	0	0.4	74.6	36.8	50.6	4.8	3.8	19.8	23.4	1.4	3.2	18.2

**Table 4:** Spearman’s correlation analysis between the daily total pollen concentration and meteorological data.

Taxa	Daily Temperature	Daily Relative Humidity	Daily Wind Speed	Daily Precipitation
Daily Total Polen	0.680 **	−0.121 **	0.137 **	−0.121
Poaceae	0.580 **	0.015	0.182 **	−0.170
Pinaceae	0.154 **	0.013	0.039	0.094
Urticaceae	0.404 **	0.088	0.247 **	−0.019
Amaranthaceae	0.311 **	0.025	0.071	0.130
*Artemisia*	0.334 **	−0.014	0.315 **	−0.363 *
Cupressaceae/Taxaceae	0.121	−0.202 **	−0.093	−0.081
*Rumex*	0.212 **	0.089	0.081	0.157
*Plantago*	0.150 *	0.227 **	0.130	−0.015
Boraginaceae	0.096	0.205 **	0.088	0.189
*Morus*	0.142	−0.126	−0.161	−0.028

** Correlation is significant at the 0.01 level (2-tailed); * Correlation is significant at the 0.05 level (2-tailed).

**Table 5:** Dominant pollen taxa in Sarıkamış and other cities.

Area/Taxa	Poaceae	Pinaceae	*Artemisia*	Amaranthaceae	Cupressaceae/Taxaceae	*Rumex*	Urticaceae	*Plantago*	Boraginaceae	*Morus*
Sarıkamış	+	+	+	+	+	+	+	+	+	+
Van, Türkiye	+	+	+	+	+	+	+	+	−	+
Bitlis, Türkiye	+	+	−	+	+	+	+	+	−	+
Kars, Türkiye	+	+	+	+	+	+	+	+	−	−
Jaipur, India	+	−	−	+	−	−	-	−	−	−
Leiden, Holland	−	−	+	+	−	+	+	+	−	−
Trentino, Italy	+	+	+	+	+	−	+	−	−	−
Mardin, Türkiye	+	+	+	+	+	−	+	+	−	+
Konya, Türkiye	+	+	−	+	+	−	+	+	+	−
Madrid, Spain	+	+	+	+	+	−	+	−	−	−
Fucnhal City, Portugal	+	+	+	+	+	+	+	+	−	−

## Data Availability

Data are contained within the article.
